# Enhancing Hip Arthroplasty Outcomes: The Multifaceted Advantages, Limitations, and Future Directions of 3D Printing Technology

**DOI:** 10.7759/cureus.60201

**Published:** 2024-05-13

**Authors:** Wael Barakeh, Omar Zein, Maya Hemdanieh, Bshara Sleem, Mohamad Nassereddine

**Affiliations:** 1 Orthopedic Surgery, American University of Beirut, Beirut, LBN

**Keywords:** 3d model, three-dimensional printing, surgery, orthopedic, hip arthroplasty

## Abstract

In the evolving field of orthopedic surgery, the integration of three-dimensional printing (3D printing) has emerged as a transformative technology, particularly in addressing the rising incidence of degenerative joint diseases. The integration of 3D printing technology in hip arthroplasty offers substantial advantages throughout the surgical process. In preoperative planning, 3D models enable meticulous assessments, aiding in accurate implant selection and precise surgical strategies. Intraoperatively, the technology contributes to precise prosthesis design, reducing operation duration, X-ray exposures, and blood loss. Beyond surgery, 3D printing revolutionizes medical equipment production, imaging, and implant design, showcasing benefits such as enhanced osseointegration and reduced stress shielding with titanium cups. Challenges include a higher risk of postoperative infection due to the porous surfaces of 3D-printed implants, technical complexities in the printing process, and the need for skilled manpower. Despite these challenges, the evolving nature of 3D printing technologies underscores the importance of relying on existing orthopedic surgical practices while emphasizing the need for standardized guidelines to fully harness its potential in improving patient care.

## Introduction and background

In the dynamic landscape of modern medicine and surgery, the evolution of technology has propelled the field into new frontiers of innovation. One advancement is the application of three-dimensional printing (3DP) - a transformative technology with far-reaching implications, particularly in the realm of orthopedic surgery. The integration of 3D printing in medicine has revolutionized surgical approaches, providing unprecedented opportunities for customization and precision [1-4].

The incidence of degenerative joint diseases, including hip osteoarthritis and necrosis of the femoral head, has surged in recent decades, aligning with the aging global population. Total hip arthroplasty procedures stand as effective interventions for end-stage joint degenerative diseases, offering relief from pain and restoration of a functional range of motion [2,5,6]. As the number of primary arthroplasties has increased, complications necessitating revision surgery increase too, such as periprosthetic osteolysis, aseptic loosening, periprosthetic fractures, and periprosthetic infections [2,7,8]. This escalation in demand for joint reconstructions has underscored the critical need for innovative solutions that go beyond the limitations of traditional approaches [9].

Therefore, the emergence of 3D printing in the orthopedic landscape has been of importance. By leveraging advanced imaging techniques, such as computed tomography (CT) scans, surgeons can now obtain detailed three-dimensional reconstructions of the patient's hip joint [10]. Its ability to produce patient-specific implants has positioned 3D printing as a key-changer in addressing the unique anatomical variations among individuals [1,11-13]. Recognizing the additive value of this technology, the present narrative review is conceptualized to explore the advancements, challenges, and future directions of 3D printing integration in the context of hip arthroplasty.

## Review

Advantages related to the surgery 

The use of 3D models in total hip replacement surgeries provides several significant advantages. The use of 3D printing in orthopedic surgeries proved to be more beneficial preoperatively, intraoperatively, and even postoperatively when compared to surgeries done without the aid of 3D printing techniques.

Preoperative

3D models serve as invaluable tools for preoperative planning, allowing surgeons to deeply analyze intricate cases. This detailed approach to planning allows the surgeon to simulate the fracture reduction process preoperatively, further refining the surgical strategy. Such meticulous preoperative assessments using 3D models not only facilitate accurate implant selection but also optimize the determination of the cup size, position, screw placement, and the need for reaming [9,10,14]. In the realm of complex revision hip arthroplasty, the employment of life-sized 3D models grants surgeons the ability for precise surgical simulations. This results in enhanced accuracy across multiple fronts including cup, augment, and buttress sizing, as well as cage templating and screw trajectory optimization, thereby reducing the risk of intraoperative neurovascular injury [10]. Furthermore, in the context of surgically applied anatomy, 3D models have proven to be more cost-effective compared to cadavers. These models enable trainers to illustrate the presence of pathology, an advantage not present in cadaveric training [15]. A study found that using 3D-printed models for preoperative examination of patient bone fractures offered a clearer understanding of the fracture patterns. This was in comparison to solely relying on 2D and 3D reconstructions viewed on a screen. The 3D models accurately represented joint fragmentations and the patterns of the articular surface, which assisted in the processes of reduction and fixation [16]. The intricate nature of 3D models plays a pivotal role in surgical readiness. It helps surgeons identify and classify bony deficiencies or fractures, presenting a clear advantage over traditional imaging techniques in understanding abnormal bone anatomy [1,9,17-19].

In their retrospective cohort study, Aprato and his team contrasted 3D CT scans with traditional CT scans to identify pelvic discontinuity in 56 patients who were receiving revision total hip replacements [20]. The initial surgical reports indicated pelvic discontinuity (PD) in nine of the 56 patients with type 3 acetabular bone defects. X-ray analysis by the first observer identified PD in 27 cases and the second observer in 21 cases. Standard CT scans showed PD in 25 cases for the first observer and 24 for the second. Using 3D models, both observers agreed on PD in 11 patients. Notably, the nine patients with PD reported in their initial surgical reports were confirmed to have PD in both CT scans and 3D models. Their findings showed that 3D models were more specific in detecting defects in the acetabulum, exhibiting flawless consistency among observers. They proposed that 3D printing technology could lead to more accurate diagnoses and better-informed management decisions compared to standard radiographic methods [20]. Moreover, Hughes et al. showed that the 3D printing technique enhances the estimation and treatment of complex pelvic deformities with greater precision. Models of actual size contributed to accurate operation simulations, facilitating improved preoperative planning and anatomical understanding. The accuracy and cost-effectiveness of this approach are likely to prove extremely valuable as a tool in clinical practice [15]. For patients, these models demystify complex acetabular fractures, making the consent process more comprehensible. This enhances the satisfaction of patients and their families [1,2,14,21]. Childs conducted a comparison between standard human hip models and 3D-printed models during patient consultations for arthroscopic hip surgery treating femoroacetabular impingement (FAI). The study revealed that the use of 3D-printed models led to an improved understanding and retention of information among patients [22].

Intraoperative

The value of 3D models extends beyond surgery preparation. They pinpoint the optimal surgical strategy, which then becomes the foundation of the surgeon's approach [21]. Such preparedness invariably cuts down on intraoperative decision-making time and ensures that placements are precise [23,24]. Mistakes, especially those arising from inexperience or less-than-optimal surgical techniques, are substantially minimized [17,24]. In a related study, using a full-size 3D-printed model was found to influence surgeons’ selection of preoperative locking plates, notably aiding less experienced surgeons in assessing complex fractures [25]. In addition, the success rates of surgeries have shown improvement when 3D-printed devices are utilized in the preoperative phase [21,26].

The 3D models also aid in precise prosthesis design, enabling the manufacturing of individualized prosthetic implants [14]. Their effectiveness has been documented for procedures like periacetabular osteotomies in hip dysplasia and predicting outcomes of scoliosis corrective surgery [27]. At Mayo Clinic, a bilateral total hip replacement was successfully conducted on a dwarfism patient who was too small for standard implants. The surgeon created a 3D-printed model of the patient's hip, and based on this model, a custom-made implant was manufactured. This tailored implant was then utilized in the joint replacement procedure [28]. Despite being relatively new to the market with only short-term results and higher costs, custom-made 3D printed implants, particularly acetabular components, are becoming increasingly favored [29]. A study conducted in Belgium has demonstrated that the aMace 3D-printed implant was significantly cost-effective in the revision arthroplasty of Paprosky-type 3B acetabular defects, compared to custom three-flanged acetabular components [30].

On the educational front, 3D models improve the learning experience for surgeons, medical students, and physicians alike, elevating skill development in orthopedic surgery [14]. The use of 3D printing in orthopedics was emphasized in a study that underlined its comprehensive utility in understanding native anatomy through both visual and tactile stimuli. They noted its significant value in the education and training of students, trainees, and surgeons and also in patient education [1]. The intraoperative advantages of using 3D-printed prostheses and navigation templates are manifold, including reductions in operation duration, X-ray exposures, and minimizing potential damage to the femoral epiphysis [17,21,24,26]. 

Furthermore, 3D models contribute to effective reductions in the neck shaft angle, trimming down surgery time, and limiting both intraoperative and postoperative blood losses [15,23,27]. Specific applications, like 3D-printed guide plates for core decompression, have shown better outcomes compared to traditional techniques, particularly in areas like reducing fluoroscopy time and intraoperative blood loss [10,23,27]. This was proved in a study that focused on assessing the effectiveness of 3D printing rapid prototyping (3DP-RP) in aiding percutaneous fixation for treating femoral intertrochanteric fractures using proximal femoral nail anti-rotation (PFNA). The study divided patients into two groups: 19 patients underwent PFNA with the assistance of 3DP-RP following computed tomography scanning, while another 20 patients received conventional PFNA treatment. The findings showed that the 3DP-RP group experienced significant reductions in surgery duration, intraoperative blood loss, and postoperative blood loss compared to the conventional surgery group [31]. 3D-printed implants, designed to align with a patient's unique anatomy, ensure a more accurate reconstruction of the hip center of rotation. This precise alignment significantly enhances the overall success and efficiency of the surgical process [8].

Postoperative

The success stories of 3D printing in orthopedics are plentiful. From treating periacetabular malignant bone tumors with personalized 3D-printed hemi pelvic prostheses to the use of 3D-printed integrated prostheses for acetabular malignancies, the results are promising [23]. Postoperative results from surgeries that employed 3D printing show improved Harris Scores after total hip arthroplasty (THA) and reduced postoperative weight-bearing times [17,19,26]. This was evidently shown in a study that utilized 3D printing technology in THA for 74 patients, with a follow-up period of about 24 months. The outcomes, including Harris scores and the time taken for postoperative weight bearing, were better in the group that received 3D printing treatment compared to the conventional group [26]. One of the most notable advantages of 3D printing is its affordability, accessibility, and potential for distributed manufacturing, reasons for its wide-scale adoption in surgical settings [32].

Printing techniques

Several 3D printing techniques are being employed in orthopedic surgery. Six main techniques have emerged as commonly utilized in this domain. Differential naming has emerged for the different techniques. For clarity, we will be using the American Society for Testing and Materials/ International Organization for Standardization (ISO/ASTM) standards for reference [33]. These include material extrusion (fused filament fabrication and direct ink writing), vat photopolymerization (stereolithography), powder bed fusion (selective laser sintering), binder jetting (particle binding), and material jetting (inkjet printing) [34].

Fused Filament Fabrication

This falls under material extrusion and entails feeding a filament of the desired material (polymers or polymer/ceramic composite materials), melting it in a vessel through heat, and extruding it from the nozzle layer by layer to form a scaffold [35].

Vat Photopolymerization

This utilizes a single-beam laser to polymerize or crosslink a photosensitive polymer, creating thin layers that are then stacked layer by layer to construct the final structure. This method presents an alternative to material extrusion. It offers precise control over the fabrication process [36].

Powder Bed Fusion

This utilizes a high-power laser to sinter metal or ceramic powders [37]. The high-power laser irradiates the powders during the printing process, enabling their fusion into cohesive structures.

Binder Jetting

This presents an innovative approach to 3D printing. It deviates from the traditional laser-based methods. Particle binding employs a liquid binding solution to fuse particles within each layer, followed by a high-temperature sintering step to solidify the final 3D products [38]. This method provides an alternative means of achieving structural integrity. It offers versatility in material compatibility.

Material Jetting

This stands out for its ability to deposit minimal volumes of individual droplets onto a printing surface, aiming to form structures through post-printing solidification [39]. The precision afforded by inkjet printing makes it suitable for intricate structures and complex geometries.

Direct Ink Writing

This is classified under extrusion-based 3D printing. It involves the extrusion of viscous materials through nozzles by compressed gas, forming individual lines that solidify onto a build plate in a layer-by-layer fashion [40]. 

Fused deposition modeling and direct ink writing are methods that are easy to use and have been employed effectively for tissue engineering applications. Selective laser sintering and particle binding have been used to create devices for hard-tissue engineering applications. Inkjet printing has been used for tissue engineering (bioadhesives, scaffolds, and living cells) and pharmaceutical applications [34]. As for the disadvantages of these various techniques, they are mostly related to the resolution, the mechanical properties, and cost [41]. A major disadvantage is the fact that almost all of them require postprocessing [41]. Some of the main advantages and disadvantages of various 3D printing techniques are listed in Table 1.

**Table 1:** Advantages and disadvantages of 3D printing techniques

Technique	Advantages	Disadvantages
Fused filament fabrication	Adequate mechanical properties, usually does not require postprocessing, and is widely accessible and relatively inexpensive	Lower resolution, needs previous filament fabrication, and needs a very high temperature for thermal degradation
Vat photopolymerization	Precise control over the fabrication process and has high resolution	Possibility of material toxicity and relatively costly
Powder bed fusion	High-power laser that offers good control over internal microstructures and high resolution	Requires a suitable particle size and relatively costly
Binder and material jetting	Fast and precise production, low temperatures are required, and relatively inexpensive	Needs a suitable ink viscosity and not very adequate mechanical properties
Direct ink writing	High drug loading efficiency, low temperatures are required, and relatively inexpensive	Lower resolution and high risk of nozzle clogging

**Figure 1:**
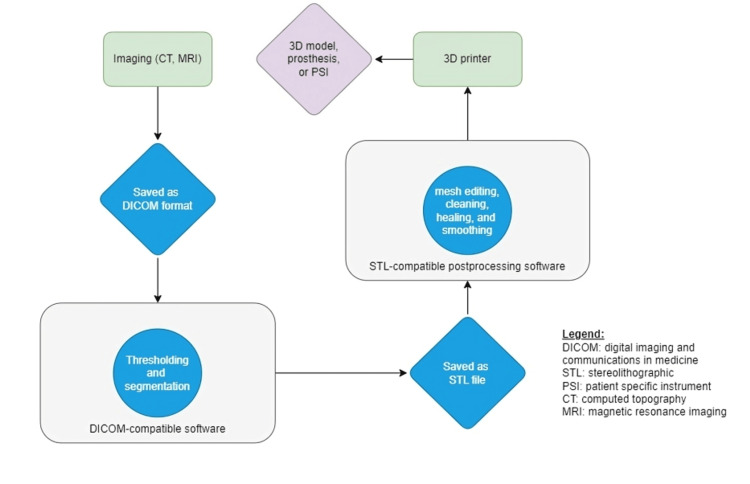
Steps of 3D printing of a model. Author credit: Wael Barakeh This image was developed by the author and not taken from anywhere else.

The diverse array of 3D printing techniques provides orthopedic surgeons with a range of options, each offering unique advantages. The choice of technique should align with the specific requirements of the intended application, considering factors such as material compatibility, structural integrity, and printing precision. Figure 1 depicts a flow diagram for the steps of 3D printing of a model.

Advantages beyond the surgery 

Prosthesis Materials and Design

The materials and design of hip prostheses have been rapidly developing. There is a continuous strive to investigate novel materials and enhance technological modalities in implant manufacturing [42]. This includes various materials, such as metals, ceramics, polymers, composites, alloys, and hybrids. This review focuses on 3D-printed metal-based hip prosthetics.

Currently used materials in hip implants, such as titanium-based alloys, cobalt-chrome alloys, and 316L stainless steel, are stiffer than bone. When a metal implant is inserted into the femur, a significant portion of the physiological load is transferred away from the more compliant surrounding bone. This altered load transmission induces underloading of the implanted femur, leading to bone remodeling. This is a process where living tissue undergoes resorption and loss of mass due to mechanotransduction sensitivity [43]. Yuan et al. suggested that increasing scaffold porosity improves biocompatibility. However, there may be a reduction in mechanical properties, such as yield strength and Young's modulus. Young's modulus is a material property that signifies its susceptibility to stretching and deformation. Porosity is viewed as inversely proportional to these properties and may result in wall thickness thinning [44].

The promise of 3D printing in orthopedic surgery extends to the development of fully porous femoral stems, with the potential to reduce stress shielding following THA and prolong implant longevity. 

Hip prostheses typically consist of two main components: the femoral component or stem and the acetabular component or Socket. Arabnejad et al. introduced a unique femoral stem design characterized by "tunable" mechanical properties, specifically in the form of a short stem taper-wedge design [45]. This innovative approach enables the microstructure of the implant to be fine-tuned to match the individual bone properties of the patient. The process involves a multi-level computerized program to adjust the stiffness of the implant in relation to the bone. The resultant specifications are then translated into a lattice mold, which is utilized in the creation of the implant through a technique referred to as "selective laser melting." This intricate procedure optimizes the 3D-printed implant to minimize resorption [1,45]. The study by Arabnejad et al. indicates that such a stem has the potential to reduce bone loss due to stress shielding by 75% [45]. These findings suggest that this innovative femoral stem design could become a crucial addition to the toolkit of orthopedic surgeons in future hip arthroplasties [1]. In addition, the ability to create customizable textured surfaces and regional stiffness can minimize irritation to overlying soft tissues, reduce stress concentration, and mitigate stress shielding [46]. 

A systematic review by Safavi et al. screened 2,530 articles and included a total of 46 studies. The review examined the potential of additive manufacturing in reducing stress shielding by incorporating higher porosity levels achievable only through this manufacturing method [47]. Three porous design strategies were identified: uniform, graded, and optimized designs. Uniform porosity involves employing a single-unit cell design repeated throughout all porous areas of the implant. Functionally graded designs refer to the systematic incorporation of defined gradients, utilizing a specified number of distinct unit cell designs distributed rationally. These distributions are based on assumptions derived from analysis and literature. Optimized porosity involves utilizing optimization algorithms to achieve the most suitable design specification. Optimized designs based on patient-specific data were found to be the most promising [47].

Titanium Cups

3D-printed titanium cups offer distinct advantages in orthopedic applications. These cups feature a porous and rough surface, theoretically promoting local vascularization and osseointegration while mitigating stress shielding due to their low elastic modulus [48]. Unlike other highly porous titanium cups produced through conventional methods, 3D-printed cups exhibit larger pore sizes and higher porosity, thereby replicating a trabecular bone-like elastic modulus. This uniqueness is achieved in monoblock implants without the need for additional coatings [49]. Consequently, 3D-printed cups can offer distinct biological advantages, including enhanced osseointegration, reduced stress shielding, and unique failure modalities, such as cracking and ion release [49]. Trabecular titanium, a specialized material used in orthopedic applications, exhibits a structure that closely resembles the trabecular bone found in the human body. This unique material can only be reproduced using high-quality additive manufacturing technology [11]. 3D printing, which is one of the many additive manufacturing technologies, is particularly suited for custom-made implants, complex geometries, and diverse surfaces, even in serial production. By contrast, conventional manufacturing techniques are primarily geared toward mass production and off-the-shelf devices due to manufacturing constraints [50]. The ability of additive manufacturing to integrate surface porosity within a monoblock implant is key to improving osseointegration and potentially reducing stress shielding by mimicking the stiffness of the periprosthetic bone [50]. It was shown that 3D-printed cups maintain acceptable micromotion, even surpassing conventional cups with lower wall thickness [48]. The combination of wall thickness and a highly porous surface contributes to the stability of 3D cups. They allow for peripheral micromotion of the sockets while providing rigidity with thicker walls [51]. Castagnini et al. in 2019 reported the results of an eight-year comparison evaluating the survival rates and reasons for revisions between trabecular titanium cups and conventional cementless cups in THA [52]. The study found that trabecular titanium cups exhibited a statistically higher survival rate compared to the control group, which used conventional cementless cups. In addition, there was a statistically lower incidence of cup aseptic loosening observed in the trabecular titanium cup group. These findings suggest that trabecular titanium cups may offer improved long-term performance and a lower risk of cup loosening in hip arthroplasty procedures [52]. These findings underscore the significant advantages of 3D-printed titanium cups in orthopedic surgery, particularly in enhancing osseointegration, reducing stress shielding, and providing stability with unique design characteristics.

Regeneration

In addition to 3D-printed customized implants, there is a noteworthy advancement in the design of orthopedic implants. These optimized implants adopt a nonporous microstructure and are constructed using materials with high cell affinity. This innovative approach promotes the proliferation of osteoblasts within the artificial joint rather than solely on the implant surface, leading to a more extensive distribution compared to traditional joint replacements. Consequently, it results in a larger contact area and increased stress resistance [53]. Enhancing the stress resistance of artificial joints holds the potential to significantly improve their long-term survival rates. This technological advancement represents a promising development in orthopedic surgery that could lead to better outcomes for patients in need of joint replacements [2].

Infection Control

In the management of artificial hip joint prosthesis infections, two-stage revisions are considered the gold standard for infection control. However, these reoperations may impose significant harm on patients due to their invasive nature. Therefore, the development of prosthetic materials with enhanced antibacterial properties can potentially reduce surgical trauma, decrease operation time, and expedite joint function recovery [24]. Zhu et al. recently employed a combination of 3D printing and antimicrobial nano-modification technology to produce zirconia ceramic implant materials with precise 3D structures and long-term wear resistance [54]. These hip prostheses precisely matched the affected area, exhibited good biocompatibility, and were sterilizable, offering the promise of improved antibacterial performance. In addition, Karaji et al. utilized electrophoretic deposition to create a silk fibroin protein solution that included calcium phosphate and vancomycin as a coating for porous titanium surfaces manufactured through additive methods [55]. This innovative approach resulted in implants with excellent antimicrobial properties and the ability to stimulate bone differentiation. Moreover, Kim et al. proposed the utilization of a 3D printed liner made from polylactic acid (PLA) as a potential solution to address the limitations of polymethylmethacrylate (PMMA) in orthopedic surgery [56]. In a comprehensive mechanical assessment, they established that PLA exhibited superior strength and ductility compared to PMMA. Furthermore, the study demonstrated the capability of PLA to facilitate controlled elution of antibiotics through the incorporation of reservoirs and microchannels [56]. Notably, the precise regulation of the liner's porosity was identified as a key factor in achieving sustained antibiotic release, a critical consideration in the context of arthroplasty-related infections. The integration of such advancements in implant materials holds the potential to improve patient outcomes by reducing infection-related complications and expediting recovery.

Disadvantages and limitations

While 3D printing technology has revolutionized many fields in orthopedics, including hip arthroplasty, it is not without its disadvantages. The utilization of 3D printing technology in orthopedics has indeed created new avenues for customized implant designs and enhanced surgical results, notwithstanding some of the limitations that can be inherent or manageable.

The risk of infection is present due to the ubiquitous dispersal of microorganisms, and 3D-printed hip implants are not exempt from posing this risk to patients and healthcare professionals. When comparing 3D printing groups to common hip replacement groups, studies have shown that the rates of postoperative infection were higher in 3D printing groups [1,19,26]. This has to do with many underlying causes, primarily the high porosity and the often-rougher finish that 3D implants have compared to their counterparts, where such surfaces offer ideal environments for bacterial growth and proliferation, ultimately leading to biofilm formation, and potentially leading to severe infections [6,57]. Periprosthetic hip infections have been estimated to occur between 0.3% and 1.7% of the time in the first two years following surgery [58]. *Staphylococcus *species are notorious for causing periprosthetic hip infections, and research is still ongoing to study fungal infections in orthopedic implants, namely, those caused by *Candida* and *Aspergillus* species [59]. Combating infections requires guidelines that enforce special sterilization techniques like gamma irradiation and autoclaving, proper preoperative skin preparation, sterile draping, the use of case-dependent antimicrobial prophylaxis, patient education, and other methods to ensure patient safety.

3D-printed hip arthroplasty also carries the burden of some technical challenges, whereby several factors like the printing time, the need for expert manpower, and the high cost that comes with it play a big role. According to Tserovski et al., the 3D printing process requires advanced computer skills and additional training, and it is a lengthy process, whereby the completion of each model requires around 12 hours [17]. In fact, the printing and overall preparation time of these models is one of the most notable limitations [6,14,24,60]. This is heavily attributed to the fact that the construction of the virtual models for the implants cannot be automated and must be handled by multidisciplinary teams that include surgeons, radiologists, and engineers [61]. After all, every patient has unique anatomical features that may prove challenging for automated algorithms to accurately capture. Another issue that can be considered a limitation is the fact that many insurance companies do not cover the use of additive manufacturing to create anatomical models [62,63]. This may have to do with the fact that additional data supporting the need for 3D printing should be established. It has been suggested by Shah et al. that when this additional data support is gathered, insurance companies can then create a dedicated Current Procedural Terminology (CPT) code for 3D printing, thus allowing for a more widespread use of this technology [64]. As for the manpower, there is a necessity for skilled users, who can either be technicians or surgeons who would have to undergo extensive training in medical modeling software modalities, which adds to the cost of this process [27,32,65], especially since the very nature of medical 3D printing often requires customization, meaning skilled users must be able to convert clinical data into digital models tailored to each patient’s unique requirements. In addition, Kumar et al. argue that implant manufacturers and producers should embed the cleaning prerequisites in the planning stage given the geometric flexibility of the implants [66]. This proactive approach can help mitigate infection risk and ensure regulatory compliance, thereby enhancing patient safety. The lack of universal standards that guide the designing and manufacturing of 3D-printed models is an additional major limitation that needs to be addressed adequately.

Guidelines

Given that 3D printing technologies are evolving by the day, they can still improve and further revolutionize orthopedics in general and hip arthroplasty in specific. In the absence of universal guidelines, it becomes imperative that healthcare professionals working with 3D printing in hip arthroplasty must rely on a combination of existing orthopedic surgical guidelines and best practices tailored to the unique aspects of 3D printing technology. Guidelines can target the biocompatibility, mechanical properties, and durability of 3D-printed materials [67]. For example, the International Organization for Standardization (IOS) issued a set of guidelines for assessing the biocompatibility of medical equipment, including those made via additive manufacturing (ISO-10993) [68]. This organization has also commented on mandating adherence to Quality Management Systems (ISO-13485) [69]. Such systems monitor the adherence of medical devices to certain requirements and specifications [70]. In addition, ASTM International provided us with guidelines pertaining to radiopacity testing of medical devices (ASTM F640-12) [71], which is very crucial in the case of 3D-printed implants to ensure their proper visibility under medical imaging techniques. In addition, many guidelines have targeted labeling and traceability, post-marketing surveillance, and education and training. Moreover, Alexander et al. raise the issue of the importance of having standard general ISO/ASTM terminology for 3D printing [33].

Many organizations are working on improving the quality of guidelines pertaining to 3D printing technologies, including the International Electrotechnical Commission (IEC), Joint Technical Committee 1 (JTC1), International Medical Device Regulatory Forum (IMDRF), and other international standards and medical device regulatory organizations like the ISO and ASTM International [62]. However, the problem remains that 3D printing in orthopedics should have its own guidelines, given its current expansion and outreach, which raise questions about many ethical and legal considerations addressing patient consent, data privacy, and customized implants. Some potential ethical considerations include the sources of cells used in bioprinting (including human embryonic stem cells and xenogeneic cells) and the use of induced pluripotent stem cells (iPSCs) that raises concerns about tumorigenicity and genetic privacy [72]. In addition, confidentiality and privacy issues may arise due to the digitization of human anatomy for bioprinting purposes. As for legal concerns, the lack of a comprehensive regulatory framework for 3D bioprinting raises concerns regarding liability and quality control. Also, concerns regarding fair distribution and social stratification can arise from the possibility that access to bioprinting technologies could widen already-existing socioeconomic gaps [72,73]. Most importantly, international harmonization is paramount when it comes to establishing specific guidelines to ensure consistency and interoperability and to further facilitate the global acceptance and market access for 3D-printed orthopedic devices. We propose the following insights as a starting point for future guidelines: the selection of biocompatible coatings that have demonstrated marked resistance to microbial colonization, the creation of implants with patient-specific textures and geometries to enhance osseointegration and lessen the chance of implant dislocation or loosening, and the employment of wearable sensors and remote monitoring to gather real-time data on patient mobility and implant stability.

Given the patient-specific nature and the lack of long-term data for 3D printing technologies, the future development of universal guidelines thus becomes essential to promote standardization and efficiency and improve the quality of care.

## Conclusions

While 3D printing in hip arthroplasty may present challenges, its profound advantages for both patients and surgeons cannot be understated. In the extensive exploration of 3D printing's applications in hip arthroplasty, a myriad of benefits emerges, ranging from meticulous preoperative planning and surgical readiness to enhanced prosthesis design and educational advancements. These advantages highlight why 3D-printed implants should be embraced. Success stories underscore its efficacy in treating complex cases, improving postoperative outcomes, and even contributing to the affordability and accessibility of orthopedic interventions on a global scale. Ultimately, these advancements are not just about innovation in surgical techniques; they are about patient safety and providing better care. Technological advancements, ongoing research, and a commitment to refining processes offer a pathway to overcome current limitations. Advancing orthopedic surgery with 3D printing technology necessitates conducting longitudinal studies to evaluate its efficacy against traditional methods, focusing on recovery, complications, and patient satisfaction. In the absence of universal guidelines, healthcare professionals engaging with 3D printing in hip arthroplasty must combine existing orthopedic surgical principles with tailored best practices. The imperative for future development of universal guidelines underscores the commitment to standardization, efficiency, and the continual improvement of care quality. While 3D printing in hip arthroplasty may present challenges, its profound advantages for both patients and surgeons cannot be understated. As the field embraces this transformative technology, the ongoing pursuit of innovation, refinement, and standardization ensures that 3D printing will play an indispensable role in shaping the future of orthopedic surgery.
